# Direct and Indirect Effects of Filamin A on Tau Pathology in Neuronal Cells

**DOI:** 10.1007/s12035-022-03121-w

**Published:** 2022-11-18

**Authors:** Stéphanie Levert, Julie Pilliod, Étienne Aumont, Sandrine Armanville, Cyntia Tremblay, Frédéric Calon, Nicole Leclerc

**Affiliations:** 1 Centre de Recherche du Centre Hospitalier de L’Université de Montréal, Montreal, Canada; 2 Département de Neurosciences, Université de Montréal, Montreal, Canada; 3 Département de Psychologie de L’Université du Québec À Montréal, Montreal, Canada; 4 Montreal Neurological Institute, Montreal, Canada; 5 Centre de Recherche du Centre Hospitalier Universitaire de Québec-Université Laval, Québec City, Canada; 6 Faculté de Pharmacie de L’Université Laval, Québec City, Canada

**Keywords:** FLNA, Tau protein, Annexin A2, F-actin, Microtubules

## Abstract

In Alzheimer disease (AD), Tau, an axonal microtubule-associated protein, becomes hyperphosphorylated, detaches from microtubules, accumulates, and self-aggregates in the somatodendritic (SD) compartment. The accumulation of hyperphosphorylated and aggregated Tau is also seen in other neurodegenerative diseases such as frontotemporal lobar degeneration (FTLD-Tau). Previous studies reported a link between filamin A (FLNA), an actin-binding protein found in the SD compartment, and Tau pathology. In the present study, we further explored this link. We confirmed the interaction of Tau with FLNA in neuroblastoma 2a (N2a) cells. This interaction was mediated by a domain located between the 157 and 383 amino acids (a.a.) of Tau. Our results also revealed that the overexpression of FLNA resulted in an intracellular accumulation of wild-type Tau and Tau mutants (P301L, V337M, and R406W) in N2a cells. Tau phosphorylation and cleavage by caspase-3 but not its aggregation were increased upon FLNA overexpression in N2a cells. In the parietal cortex of AD brain, insoluble FLNA was increased compared to control brain, but it did not correlate with Tau pathology. Interestingly, Tau binding to microtubules and F-actin was preserved upon FLNA overexpression in N2a cells. Lastly, our results revealed that FLNA also induced the accumulation of annexin A2, a Tau interacting partner involved in its axonal localization. Collectively, our data indicated that in Tauopathies, FLNA could contribute to Tau pathology by acting on Tau and annexin A2.

**Supplementary Information:**

The online version contains supplementary material available at 10.1007/s12035-022-03121-w.

## Introduction

Alzheimer disease (AD) is the most common type of dementia that is affecting more than 44 million people worldwide. Defining the causes of AD and understanding what triggers it remain a challenge. In AD, Tau, an axonal microtubule-associated protein, becomes hyperphosphorylated, detaches from microtubules, accumulates, and self-aggregates in the somatodendritic (SD) compartment [1–3]. Abnormal deposition of misfolded and aggregated Tau forms neurofibrillary tangles (NFTs), which are correlated with the onset of cognitive impairment in patients [4–10].

Filamins are actin-binding proteins that contribute to the link between the extracellular matrix and the cytoskeleton [11–15]. Human filamins present three isoforms, FLNA, FLNB, and FLNC [13]. The FLNA isoform is mostly found in the human brain and has several binding partners interacting with its C-terminal rod2 region [16–18]. FLNA is involved in several signaling pathways implicated in cell adhesion and migration through its partners, such as GTPases, kinases, and transmembrane receptors [13, 19–21]. FLNA was also shown to affect the degradation of a protein when interacting with it. For example, this was demonstrated for the cystic fibrosis transmembrane conductance regulator (CFTR) [22]. Growing evidence indicate that FLNA could be involved in AD. In recent proteomics studies, the protein levels of FLNA as well as its aggregation were found to be higher in AD than in control brain [23–25]. This is consistent with previous studies reporting that presenilin-1 (PS1) mutations, which are linked to early-onset of AD, were associated with an increase FLNA expression [26, 27]. Aβ peptide that is believed to play a central role in AD was reported to alter the conformation of FLNA. This change of conformation promoted the binding of FLNA to the α7 nicotinic acetylcholine receptor (α7nAChR), which resulted in Tau hyperphosphorylation by the activation of the kinases ERKs and c-Jun N-terminal kinase 1[28–30]. Furthermore, altered FLNA by Aβ could also interact with toll-like-receptor 4 (TLR4) causing its persistent activation that led to excessive inflammatory cytokine [31]. A potential direct link between FLNA and Tau pathology could also exist in AD brain. FLNA was found in NFTs by using immunocytochemistry and mass spectrometry (MS) [32, 33]. These data suggest that FLNA could be part of a convergent pathway leading to Aβ and Tau pathologies in AD.

In a more recent study, a direct link between FLNA and Tau pathology was established in progressive supranuclear palsy (PSP) [34]. PSP is a Tauopathy characterized by the accumulation and aggregation of Tau isoforms containing four microtubule-binding domains [35]. Tsujikawa et al., by using proteomics and genomics analyses, revealed that PSP was associated with FLNA genetic variation and the increased protein levels of FLNA [34]. Furthermore, they demonstrated that FLNA induced an accumulation of phosphorylated and insoluble Tau in HEK 293 cells and in murine brain.

The above studies established a link between FLNA and Tau pathology. However, important information on this link remains unexplored. In the present study, five points were investigated: (1) Does FLNA induce the cytoplasmic accumulation of Tau mutants linked to FTLD as it does for wild-type Tau? (2) Does FLNA affect the cleavage of Tau by caspase-3? (3) Does FLNA alter the binding of Tau to microtubules and F-actin? (4) Does FLNA insolubility correlate with Tau pathology in AD brain? (5) Does FLNA induce the cytoplasmic accumulation of proteins other than Tau such as annexin A2, known to interact with both Tau and FLNA? [36–38] Our results revealed that FLNA could contribute to mutated and wild-type Tau pathology by altering Tau protein levels, phosphorylation, and cleavage by caspase-3 and by inducing the accumulation of its partner annexin A2. However, Tau was still able to bind to microtubules and F-actin upon FLNA overexpression. Lastly, no correlation between FLNA insolubility and Tau pathology was noted in the parietal cortex of post-mortem AD brain samples indicating that the contribution of FLNA to Tau aggregation could depend on the brain region.

## Materials and Methods

### Human Brain Tissues

Parietal cortex (precuneus area) samples were obtained from participants from the ROS (Religious Order Study), a longitudinal clinical and pathological study of aging and dementia, as described previously [39–43]. Participants received a clinical diagnosis of either dementia, mild cognitive impairment (MCI) or no cognitive impairment (NCI) (*n* = 20 for each group) at the time of death by a neurologist, blinded to all post-mortem data (Table 1). The neuropathological diagnosis used here for comparative analyses was based on the ABC scoring method found in the revised National Institute of Aging – Alzheimer’s Association (NIA-AA) guidelines for the neuropathological diagnosis of AD [40, 44]. Individuals classified as “controls” (*n* = 20) had no or low levels of AD neuropathological changes while those classified as “AD” (*n* = 20) presented either intermediate or high levels of AD neuropathological changes.

**Table 1:** Patient characteristics

Characteristics	NCI	MCI	Dementia	Statistical analysis
*N*	20	20	20	n/a
Age	87.1 (5.82)	87.1 (5.20)	87.3 (4.85)	*F*(57) = 0.01; *p* = 0.99
Men (%)	20	45	35	*χ*^2^(2) = 2.85; *p* = 0.24
APOE ε4 carriers (%)	30	30	35	*χ*^2^(2) = 0.15; *p* = 0.93
Years of education	18.5 (3.59)	18.6 (3.00)	17.5 (3.00)	*F*(57) = 0.69; *p* = 0.51
MMSE score	27.2 (1.81)	25.5 (3.07)	15.8 (7.89)	*F*(57) = 30.16; *p* < 0.001*
Post-mortem interval, hours	7.45 (5.52)	7.82 (5.22)	7.78 (4.81)	*F*(55) = .03; *p* = 0.97
Cerebellar pH	6.35 (0.342)	6.32 (0.279)	6.31 (0.459)	*F*(57) = .07; *p* = 0.93
Neuropathological AD (%)	45	60	85	*χ*^2^(2) = 7.03; *p* = 0.03*
Episodic memory CS	0.266 (0.483)	− 0.431 (0.725)	− 1.97 (1.19)	*F*(56) = 35.92; *p* < 0.001^2^
Semantic memory CS	− 0.210 (0.633)	− 0.349 (0.607)	− 1.50 (1.23)	*F*(56) = 12.92; *p* < 0.001*
Working memory CS	− 0.245 (0.460)	− 0.428 (0.665)	− 0.963 (0.847)	*F*(56) = 6.08; *p* = 0.004*
Processing speed CS	− 0.362 (0.798)	− 0.890 (0.898)	− 2.17 (0.849)	*F*(56) = 23.99; *p* < 0.001*
Visuospatial ability CS	− 0.208 (0.723)	− 0.141 (0.657)	− 1.08 (0.952)	*F*(56) = 8.78; *p* < 0.001*
General cognition CS	− 0.027 (0.375)	− 0.430 (0.447)	− 1.66 (0.885)	*F*(56) = 38.02; *p* < 0.001*

*NCI*, no cognitive impairment; *MCI*, mild cognitive impairment. *p < 0.05 between AD and both cognitively normal and MCI; ^2^*p* < 0.05 between all groups

Post-mortem samples (100 mg) of the parietal cortex obtained from all participants were homogenized (first in Tris-buffered saline [TBS]), sonicated, and centrifuged sequentially to generate (i) a TBS-soluble fraction (containing intracellular and extracellular soluble proteins), (ii) a detergent-soluble fraction (containing detergent-soluble membrane-bound proteins), and 2 detergent-insoluble fractions (containing insoluble proteins) either (iii) solubilized in FA or (iv) extracted using SK as previously described [43]. Equal amounts of proteins per sample (15 µg of total proteins per lane) were added to Laemmli’s loading buffer and heated to 95 °C for 5 min (min).

### Cell Culture and Transfection

N2a cells were purchased from ATCC (number CCL131TM, Manassas, VA, USA) and were cultured in minimal essential medium with Earle’s salt, nonessential amino acids supplemented with l-glutamine, sodium pyruvate, and sodium bicarbonate (Wisent Life Sciences, Saint Bruno, QC, Canada), and with 10% fetal bovine serum premium (Wisent Life Sciences) at 37 °C in a humidified 5% CO_2_ incubator. For transfection, N2a cells were plated at a density of 9 X 10 5 cells in 35-mm Petri dishes and grown overnight to 60% confluency. JetOPTIMUS (Polyplus Transfection, New York, NY, USA) was used for co-transfection of N2a cells. For each dish, 0.5 µg of each plasmid DNA was mixed with 200 µL of JetOPTIMUS buffer and 1 µL of JetOPTIMUS. This mixture was incubated for 10 min and was then added to each dish. Forty-eight hours (h) after transfection, the cells were lysed for immunoblotting.

### Plasmids and Antibodies

The plasmids pCR-CMV Flag Tau 0N4R (Flag-4RTau) and pEGFP-C1 (GFP-empty) were previously described [45]. The plasmids pcDNA3-myc-FLNa WT (myc-FLNA) and pcDNA3-myc-empty (myc-empty) were purchased from Addgene (#8982, Cambridge, MA, USA). Flag-4RTau-P301L, Flag-4R-Tau-R406W, and Flag-4R-Tau-V337M were obtained by single site-directed mutagenesis from Flag-4RTau. The plasmid GFP-MAP2c was obtained by cloning the rat MAP2c sequence into the pEGFP-C1. The GFP-annexin A2 and GFP-VAMP8 plasmids were purchased from Addgene (#42,311 for GFP-VAMP8 and #107,196 for annexin A2). The pEGFP-C1 Tau0N4R MBD-CT (157–383) (GFP-TauMBD-CT) and pEGFP-C1 Tau0N4R NT (1–156) (GFP-TauNT) were obtained by single site-directed mutagenesis from pEGFP-C1 Tau0N4R (GFP-4RTau).

For immunoblotting and immunoprecipitation, the following antibodies were used: total Tau (1:40,000, number A0024, Dako, Santa Clara, CA, USA), ɣ-actin (1:10,000, #Sc-65635, Santa Cruz, Dallas, TX, USA), c-myc (1:2000, #626,802, BioLegend, San Diego, California, USA), FLNA (1:2000, #4762, Cell Signaling Technology, Danvers, MA, USA), Tau-1 (1:10,000, #MAB3420, EMD Millipore, Etobicoke, ON, Canada), AT180 (1:100, #MN1040, Thermo Fisher Scientific, Waltham, MA, USA), phospho-S199-202 (1:1000, #44-768G, Invitrogen, Carlsbad, CA, USA), phospho-T205 (1:500, #44-738G, Invitrogen, Carlsbad, CA, USA), phospho-FLNA (Ser2152) (1:2000, #4761, Cell Signaling Technology, Danvers, MA, USA), Myc (1:1000, #9E1, ChromoTek, Planneg, Germany), GFP (1:1000, #3H9, ChromoTek Inc., Hauppauge, NY, USA), TauC3 (1:1000, #AHB0061, Thermo Fisher Scientific, Waltham, MA, USA), acetylated tubulin (1:2000, #T6793, Sigma, Billerica, MA, USA), tubulin (1:5000, #T9026, Sigma, Billerica, MA, USA), and annexin A2 (H-5, #SC-48397, Santa Cruz, Dallas, TX, USA).

### Cell Lysis

Cells were washed twice with PBS and once with PBS containing 0.5 M NaCl and lysed in fresh lysis buffer containing Tris 50 mM, NaCl 300 mM, Triton X-100 (0.5%), a protease inhibitor mixture (Complete EDTA-free, Roche Diagnostics, Indianapolis, IN), and a phosphatase inhibitor mixture (PhosSTOP, Roche Diagnostics), and then incubated on ice for 20 min. For SDS-PAGE, the cell lysates were centrifuged at 12,000 rpm for 20 min at 4 °C. Proteins were quantified using Bio-Rad DC Protein assay (Bio-Rad Laboratories Ltd., Mississauga, ON, Canada). Equal amounts of proteins were mixed with Laemmli buffer and boiled for 5 min at 95 °C.

### Western Blot of Cell Lysates

The mixtures of proteins boiled in Laemmli buffer were separated on polyacrylamide gels and transferred to a nitrocellulose membrane. Immunoblotting was performed as previously described [45]. Briefly, the nitrocellulose membranes were blocked in 5% nonfat dry milk and TBS-T (Tris-buffered saline with 0.2% Tween-20 (Sigma)) for 1 h and then were incubated with the primary antibodies overnight or 1h30 at room temperature. Then, the membranes were washed with TBS-T. All the secondary antibodies used to reveal the primary antibodies were coupled with horseradish peroxidase and were purchased from Jackson ImmunoResearch (West Grove, PA, USA). For quantification of the immunoreactive bands, western blotting image acquisition was performed using a ChemiDoc MP system (Bio-Rad Laboratories, Hercules, CA, USA) and densitometry analysis was done with the Image Laboratory software (version 5.0, Bio-Rad Laboratories).

### Western Blot of Human Brain Fractions

The mixtures of proteins boiled in Laemmli buffer were subjected to SDS polyacrylamide gel electrophoresis and electroblotted onto PVDF membranes (GE Healthcare, Mississauga, ON, Canada). The membranes were blocked in 5% nonfat dry milk and 0.5% bovine serum albumin in 10 mM phosphate-buffered saline for 1 h. They were then incubated with the appropriate primary and secondary antibodies and bands were visualized by chemiluminescence (Luminata Forte, Millipore, Etobicoke, ON, Canada). Image acquisition and analysis were performed with a KODAK Image Station 4000MM (Molecular Imaging Software version 5.3.4, Carestream, Woodbridge, CT).

### RNA Extraction and qRT-PCR

Extraction of RNA from N2a cells was performed using an RNeasy Plus Mini Kit (Qiagen, Toronto, ON, Canada), following the manufacturer’s instructions. The measurement of Tau DNA content by real‐time quantitative PCR was performed using on a QuantStudio™ 7 flex system instrument, TaqMan® Reagents, and analyzed with QuantStudio Real-Time PCR software (Applied Biosystems, Foster, CA, USA).

### GFP-Trap and Myc-Trap Immunoprecipitation

N2a cells transfected with either GFP-TauWT, GFP-Tau-MBD-CT, GFP-TauNT or pEGFP-C1 empty were washed once with PBS and once with PBS containing 0.5 M NaCl. The cell pellets were lysed in fresh lysis buffer containing imidazole 3 mM pH 7.4, sucrose 250 mM, and a protease inhibitor mixture (Complete EDTA-free, Roche Diagnostics, Indianapolis, IN). Syringes 25G and 27 1/2G were used for lysing the cells. The cell lysates were centrifuged at 3000 rpm for 10 min to remove cell debris. Proteins were quantified using Bio-Rad DC Protein assay (Bio-Rad Laboratories Ltd., Mississauga, ON, Canada). The total protein concentration in the cell lysate was adjusted to 620 µg/mL and then, the cell lysate was centrifuged at 44,000 rpm for 1 h. The pellet was detached with 1 mL of HBS solution containing HEPES 20 mM pH 7.2, NaCl 150 mM, sucrose 250 mM, and the Roche Diagnostics protease inhibitor mixture and sonicated 1–2 pulse of 1–2 s at 30% amplitude. A total of 20 µL of control magnetic beads and 20 µL magnetic beads coupled with anti-GFP Nanobody (ChromoTek) were washed with HBS-T (HBS solution with 0.02% Triton). The resuspended pellet was incubated for 2 h with control magnetic beads at 4 °C and then incubated for 2 h with GFP magnetic beads at 4 °C. Twenty microliters of cell lysate before ultracentrifugation and after sonication and the supernatant of GFP magnetic beads were collected and mixed with Laemmli buffer. These samples were identified as the Input1% fraction, Input2% fraction, and the unbounded fraction for immunoblotting. For the elution, using a magnetic rack, the GFP magnetic beads were washed with HBS, mixed with 2X Laemmli buffer (Tris 140 mM pH 6.8 and 7.5% SDS), and boiled for 10 min at 95 °C. Using a magnetic rack, the mixtures were collected, and the magnetic beads were washed with sterile water. Immunoprecipitates were separated using precast 4–20% gradient gels and immunoblotting was performed as described in the “Western Blot of Cell Lysates” section. For the myc-Trap experiments, the lysates of N2a cells co-transfected with either GFP-TauWT and Myc-FLNA, GFP-Tau-MBD-CT and Myc-FLNA, GFP-TauNT and Myc-FLNA or pEGFP-C1 and Myc-FLNA were used and were processed as described for the GFP-trap.

### Immunofluorescence and Airyscan Imaging Setup

N2a cells grown on coverslips coated with poly-d-lysine (Mandel Scientific Company Inc., Guelph, ON, Canada) were fixed in 4% PFA for 10 min at 37 °C and permeabilized with 0.2% Triton X-100 in PBS for 5 min. Coverslips were blocked with 5% normal goat serum (NGS, Invitrogen) in PBS. Then, coverslips were incubated with primary antibodies for 1 h at room temperature. After 3 washes in PBS, coverslips were incubated with the secondary antibodies coupled to an Alexa chromophore for 1 h at room temperature (Jackson ImmunoResearch). Coverslips were then washed in PBS and mounted in fluoromount G (SouthernBiotech, Cedarlane). Airyscan images were acquired using an inverted Zeiss LSM900 microscope (Zeiss, Germany) equipped with the Airyscan 2 module. All images were acquired using a Plan-Apochromat 40 × /1.3 Oil DIC (UV) VIS-IR M27 objective (Zeiss, Germany) and a GaAsP-PMT Airyscan detector (Zeiss, Germany). For excitation, a 640-nm (5mW), a 561-nm (10mW), and a 488-nm (10mW) diode laser were used for Alexa Fluor 647, Alexa Fluor 568, and GFP, respectively. Bandwidth detections of 644–700 nm, 569–626 nm, and 491–548 nm for AF647, AF568, and GFP, respectively, were set. Acquisition of each channel was done sequentially in a bidirectional mode and in 8bit. Pixel size was automatically set for SR mode (46 nm/pixel), with a dwell time between 0.79 and 1.57 µs/pixel (depending on the zoom factor, indicated in legends). ZStack were acquired with a 0.5 µm step range to image the entire volume of a cell. Images were acquired using the Zen Blue software V3.5 (Zeiss, Germany). Airyscan images were 3D deconvolved using the standard mode of the Airyscan Joint Deconvolution module of the Zen Blue software.

### CisBio Tau Aggregation Kit

Homogeneous Time Resolved Fluorescence (HTRF) experiments were performed with the CisBio Tau aggregation kit (CisBio GmbH, Berlin, Germany) following the manufacturer’s indications with some modifications. The cell lysates were collected as described in the “Cell Lysis” section. Four dilutions of the cell lysates were used with a total protein concentration of either 0.0025 ng/µL, 0.035 ng/µL, 0.005 ng/µL or 0.0125 ng/µL. Ten microliters of a mixture of the same amount of each conjugated antibody was added to 10 µL of cell lysates. The mixture was incubated for 20 h at room temperature and read with a EnVision plate reader at 665 nm and 620 nm to obtain the ratio of the time resolved FRET (665 nm) on the donor emission (620 nm) that is artifact-free.

### Statistical Analysis

For the densitometry analysis of the western blots of the N2a cell lysates, a paired *t* test was used. The statistical analysis was performed using the GraphPad Prism 8 software and *p* < 0.05 was considered significant. Results from the densitometry analysis of the brains fractions were analyzed using RStudio version 2021.9.1.372 (RStudio Team, 2020) with *p* < 0.05 used as a statistical threshold. We performed an ANCOVA comparing levels of insoluble FLNA between non-AD subjects and subjects with AD with age, sex, APOE ε4 carrier status, and study batch as covariates (RStudio Team, RStudio: Integrated Development for R, RStudio, Boston, MA, 2020, http://www.rstudio.com/). The association between insoluble FLNA and total insoluble Tau concentrations and the ratio of phosphorylated insoluble Tau over total insoluble Tau were tested using partial correlations with age, sex, and study batch as covariates. Insoluble FLNA levels adjusted for age, sex, and study batch were then correlated with Braak stages with a Spearman correlation.

## Results

### FLNA Induces Wild-Type and Mutated Tau Accumulation in N2a Cells

**Fig. 1:**
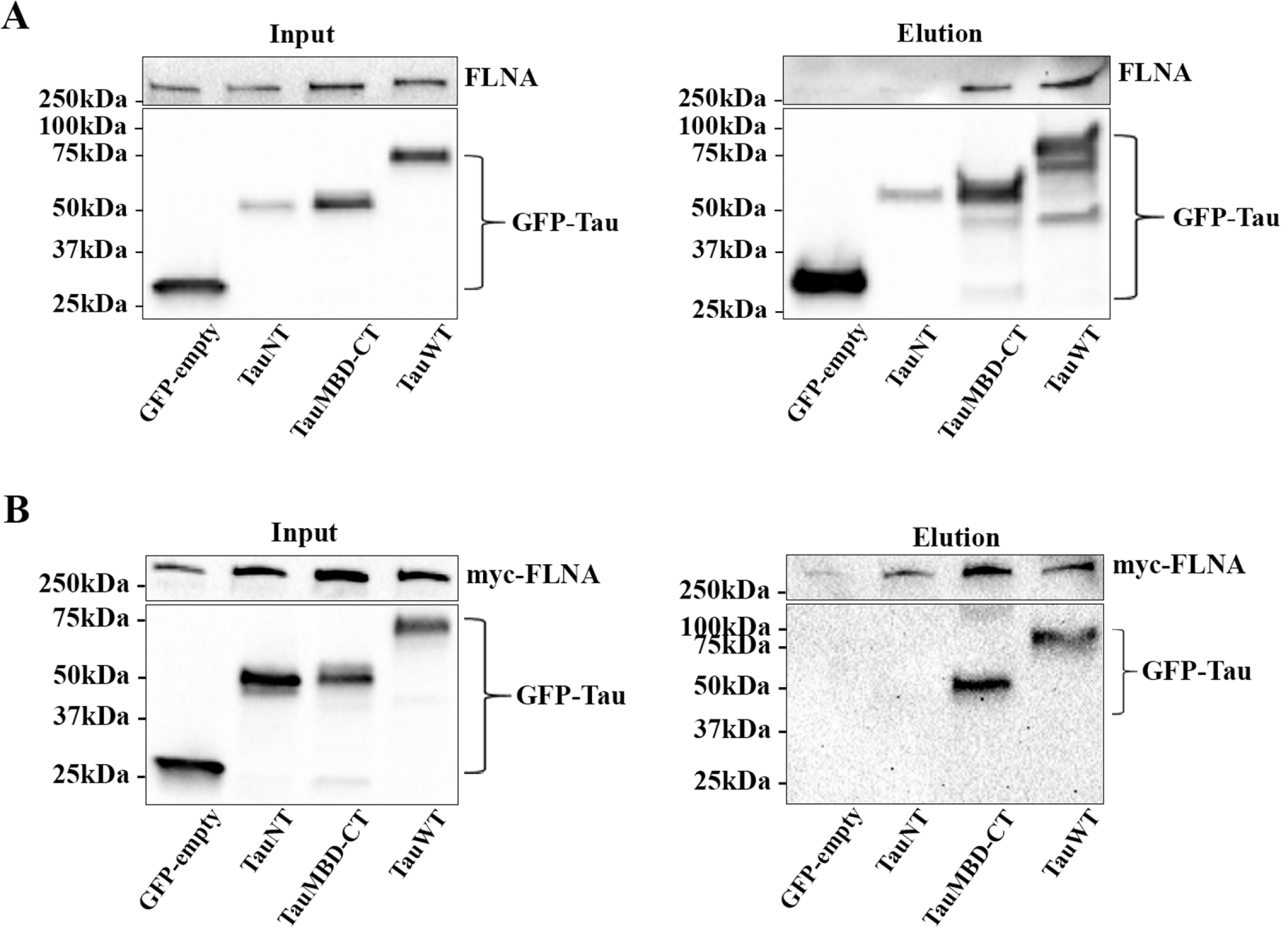
Co-immunoprecipitation of FLNA and Tau in N2a cells. GFP-trap was used to co-immunoprecipitate FLNA by Tau and myc-trap for co-immunoprecipitate Tau by FLNA. For GFP-trap, N2a cells were transfected with either EGFP-empty plasmid, GFP-4RTau-NT plasmid (TauNT), GFP-4RTauMBD-CT plasmid (TauMBD-CT) or GFP-4RTau plasmid (TauWT) for 48 h. For Myc-trap, N2a cells were co-transfected with either GFP-empty and myc-FLNA plasmids, GFP-4RTau-NT and myc-FLNA plasmids, GFP-4RTauMBD-CT and myc-FLNA plasmids or GFP-4RTau and myc-FLNA plasmids for 48 h. **A** Western blotting analysis of the input and elution fractions obtained from the GFP-trap experiments with the antibody directed against GFP confirmed the presence of Tau in all conditions. Endogenous FLNA was found in all the input fractions but only in TauWT and TauMBD-CT elution fractions. *n* = 3. **B** Western blotting analysis of the input and elution fractions obtained from myc-trap experiments with the antibody recognizing the myc-tag confirmed the presence of myc-FLNA in all conditions. The antibody directed against GFP revealed the presence of Tau in all the input fractions but only in TauWT and TauMBD-CT elution fractions. *n* = 3

Tsujikawa K. et al. reported that Tau interacts with FLNA contributing to Tau pathology by increasing its phosphorylation and aggregation in HEK293 cells and mouse brain [34]. In the present study, the GFP-trap technology was used to detect FLNA and Tau interaction in N2a cells. When N2a cells were transfected with GFP-4RTau, endogenous FLNA was found in GFP-Tau immunoprecipitate (Fig. 1A). To determine whether the domain involved in its interaction with FLNA was located in either the N-terminal or C-terminal half of Tau, a GFP-tagged Tau construct either containing the 1–156 amino acids (a.a.) of 0N4R called TauNT or containing 157–383 a.a. called TauMBD-CT was expressed in N2a cells. A strong FLNA signal was found in GFP-TauMBD-CT immunoprecipitate indicating that Tau domain mediating its interaction with FLNA was contained between the 157 and 383 a.a. (Fig. 1A). A very weak signal was observed in the GFP-TauNT immunoprecipitate similar to that found in the GFP-empty immunoprecipitate, indicating that TauNT did not interact with FLNA (Fig. 1A). The converse experiment was carried out where we tested whether Tau could be immunoprecipitated by FLNA. For this set of experiments, the myc-trap approach was used since FLNA was fused to the myc tag. N2a cells were co-transfected with either myc-FLNA and GFP-TauWT, myc-FLNA and GFP-TauMBD-CT, myc-FLNA and GFP-TauNT or myc-FLNA and GFP-empty. TauWT and TauMBD-CT but not TauNT were found in myc-FLNA immunoprecipitate as revealed by the GFP antibody, confirming that the interaction between FLNA and Tau was mediated by a domain found in the 157–383 a.a. (Fig. 1B).

**Fig. 2:**
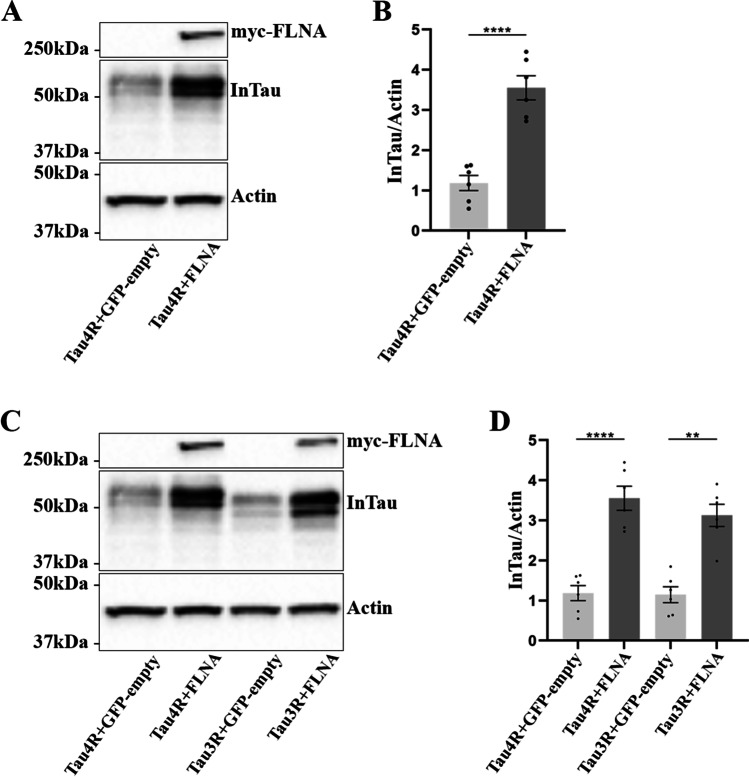
Intracellular accumulation of 3R and 4R Tau isoforms upon FLNA overexpression. N2a cells were co-transfected with either Flag-4RTau (Tau4R) and EGFP-empty plasmids, Flag-4RTau and myc-FLNA, Flag-3RTau (Tau3R) and GFP-empty plasmids or Flag-3RTau and myc-FLNA plasmids for 48 h. **A** Western blotting analysis of the cell lysate with the antibody A0024 revealed an increase of the intracellular wild-type Tau (InTau) upon the overexpression of FLNA. **B** Densitometry analysis of the A0024 signal of InTau, *n* = 6. **C** Western blotting analysis of the cell lysate with the antibody A0024 revealed an increase of 3R and 4R Tau isoforms upon the overexpression of FLNA. **D** Densitometry analysis of the A0024 signal of 3R and 4R Tau isoforms revealed a significant increase in both isoforms upon FLNA overexpression, *n* = 6. Actin was used as a loading reference and the antibody directed against the myc-tag was used to confirm the expression of myc-FLNA. InTau was normalized with the actin signal. Data represent scatter plot and mean ± SEM. Each dot corresponds to a *n*. **p < 0.01; ****p < 0.0001

**Fig. 3:**
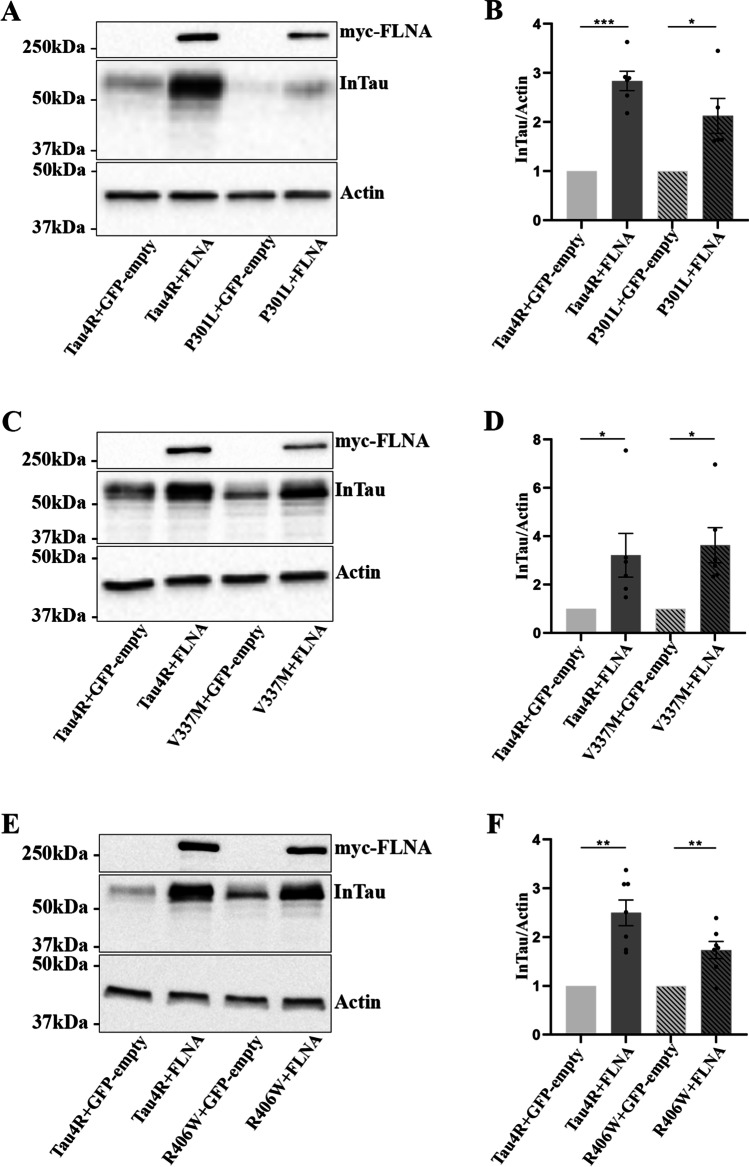
Intracellular accumulation of Tau mutants upon FLNA overexpression. N2a cells were co-transfected with either Flag-4RTau and EGFP-empty plasmids, Flag-4RTau and myc-FLNA, Flag-4R-P301L and EGFP-empty plasmids, Flag-4R-P301L and myc-FLNA plasmids, Flag-4R-V337M and EGFP-empty plasmids, Flag-4R-V337M and myc-FLNA plasmids, Flag-4R-R406W and EGFP-empty plasmids or Flag-4R-R406W and myc-FLNA plasmids for 48 h. **A**, **C**, **E** Western blotting analysis with the antibody A0024 of the cell lysate revealed a significant increase of intracellular InTau upon overexpression of FLNA for wild-type Tau and Tau mutants. Actin was used as a loading reference and the antibody directed against the myc-tag was used to reveal myc-FLNA. InTau was normalized with the actin signal. **B**, **D**, **F** Densitometry analysis of the InTau signal. For a better representation, the signal of P301L in the condition Flag-4R-P301L and EGFP-empty was reported to 1. Data represent scatter plot and mean ± SEM; *n* = 5 or 6. Each dot corresponds to a *n*. *p < 0.05; **p < 0.01; ***p < 0.001

In the previous study reporting FLNA interaction with Tau, an accumulation of Tau in HEK 293 cells and mouse brain was observed upon FLNA overexpression [34]. Based on this study, we examined the effects of FLNA overexpression on Tau protein levels in N2a cells. The protein levels of total Tau were significantly increased upon the overexpression of FLNA in N2a cells compared to cells overexpressing Tau alone as revealed by western blotting using the anti-Tau antibody A0024 (Fig. 2A, B). We also examined whether the intracellular accumulation induced by the overexpression of FLNA was dependent on Tau isoform. To test this, N2a cells were co-transfected with either Tau 0N3R (3RTau) and FLNA or Tau 0N4R (4RTau) and FLNA. The immunoblotting analysis of the cell lysates revealed a significant increase of 3R and 4R protein levels upon overexpression of FLNA (Fig. 2C, D). The aforementioned results indicated that FLNA could induce the accumulation of all Tau isoforms in N2a cells as recently reported in HEK 293 cells and mouse brain by Tsujikawa et al. [34]. Lastly, we investigated whether FLNA could increase the intracellular accumulation of Tau mutants TauP301L, TauV337M, and TauR406W found in FTLD-Tau [1]. These mutants were co-transfected with FLNA in N2a cells. An intracellular increase of all three mutants was found in N2a cell lysates upon FLNA overexpression (Fig. 3A–F). We noted that the levels of accumulation were different for these three mutated forms of Tau, some presenting a higher accumulation than others. The above observations indicated that FLNA could be involved in Tau accumulation observed in all Tauopathies.

**Fig. 4:**
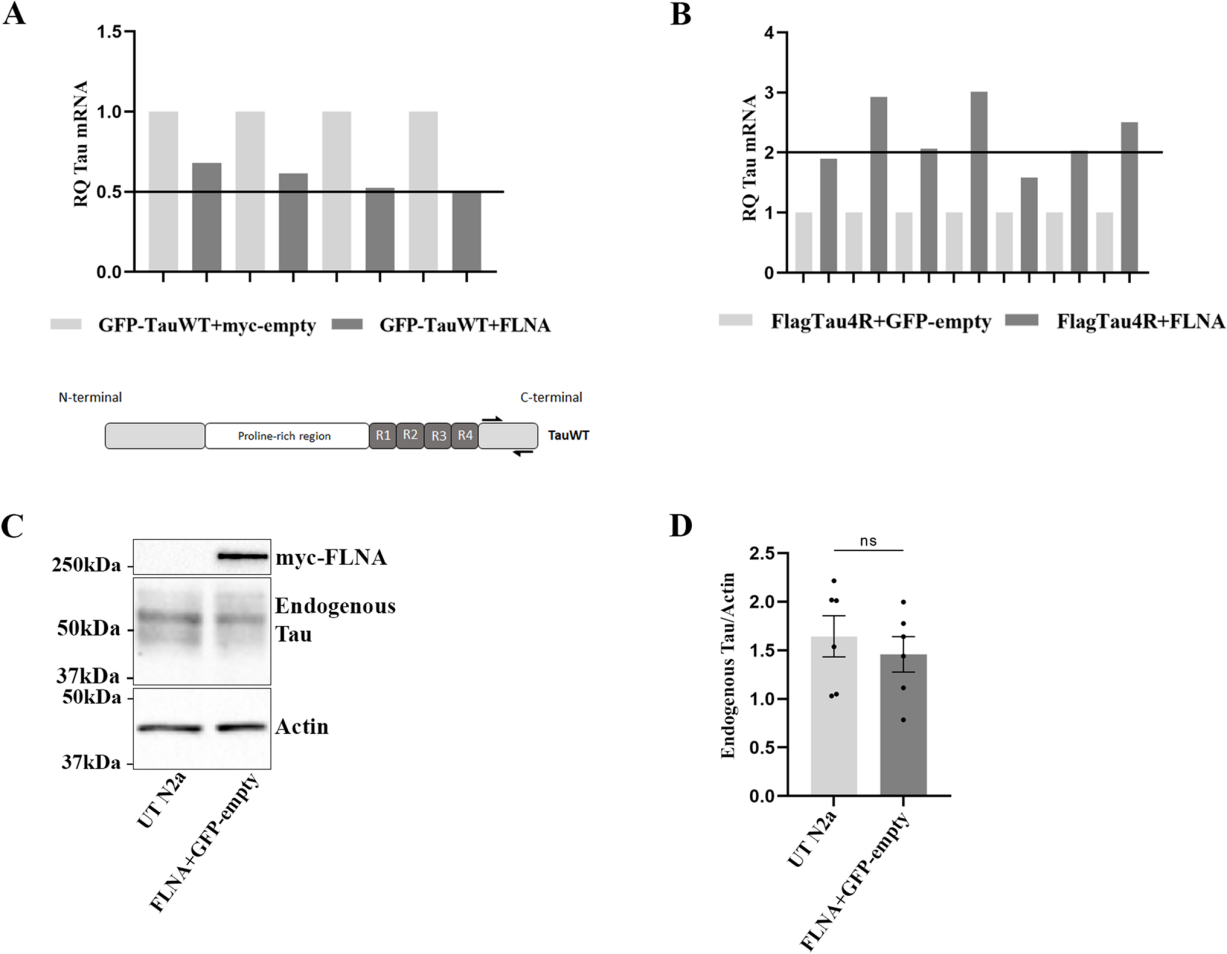
Increase of Tau mRNA is not responsible for Tau intracellular accumulation upon FLNA overexpression. GFP-4RTau plasmid (TauWT) was co-transfected with either myc-empty or myc-FLNA in N2a cells for 24 h. **A** The relative quantification (RQ) of TauWT expression was analyzed as mean ± SEM, *n* = 4. RQ corresponds to the fold change compared to the calibrators (GFP-TauWT + myc-empty). The calibrator has a RQ of 1. RQ is considered significant when there is a minimum of two-fold change (RQ > 2 or RQ < 0.5). **B** Flag-4RTau plasmid was co-transfected with either EGFP-empty or myc-FLNA plasmids for 24 in N2a cells. RQ of Tau expression of Tau4R by RT-qPCR was analyzed on 7 independent experiments. The calibrator (Flag-Tau4R + GFP-empty) has a RQ of 1. **C** N2a cells were co-transfected with myc-FLNA and GFP-empty for 48 h and untransfected cells (UT N2a) were used as controls. Western blotting analysis with the antibody A0024 of the cell lysate revealed endogenous Tau in both conditions. **D** Densitometry analysis of the A0024 signal of endogenous Tau showed no significative difference between untransfected cells and cells transfected with myc-FLNA, *n* = 6. Each dot corresponds to a *n*. *p < 0.05; **p < 0.01; ***p < 0.001

To elucidate how FLNA overexpression resulted in the accumulation of Tau in N2a cells, we examined whether FLNA induced an increase of Tau expression. Tau expression was measured by RT-qPCR in experiments where either Flag-4RTau or GFP-4RTau was co-transfected with either myc-FLNA or myc-empty. No increase of Tau mRNA was observed when GFP-Tau and FLNA were overexpressed compared to GFP-4RTau and myc-empty (Fig. 4A). In the case of Flag-4RTau, in 3 out of 7 experiments, there was a clear increase of Tau mRNA when cells were co-transfected with myc-FLNA (Fig. 4B). However, in all these experiments, an increase of Tau protein levels was observed demonstrating that the increase of Tau mRNA was not necessary to Tau accumulation upon FLNA overexpression, although it could contribute to it. The vector used for overexpression of human Tau contained the coding sequence of Tau driven by CMV promoter without any regulatory elements and promoter regions of Tau suggesting that exonic regions of Tau would be involved in the effects of FLNA on Tau expression. Such a mechanism was previously demonstrated for Tau [46]. However, the fact that no accumulation of endogenous Tau was detected upon FLNA overexpression demonstrated that the main effects of FLNA on Tau accumulation did not implicate a mechanism involving exonic regions of Tau in N2a cells (Fig. 4C, D). These results demonstrated that FLNA-induced accumulation of Tau occurred through a mechanism other than increasing its expression.

### FLNA Overexpression Increases Tau Phosphorylation and its Cleavage by Caspase-3 but not its Aggregation

**Fig. 5:**
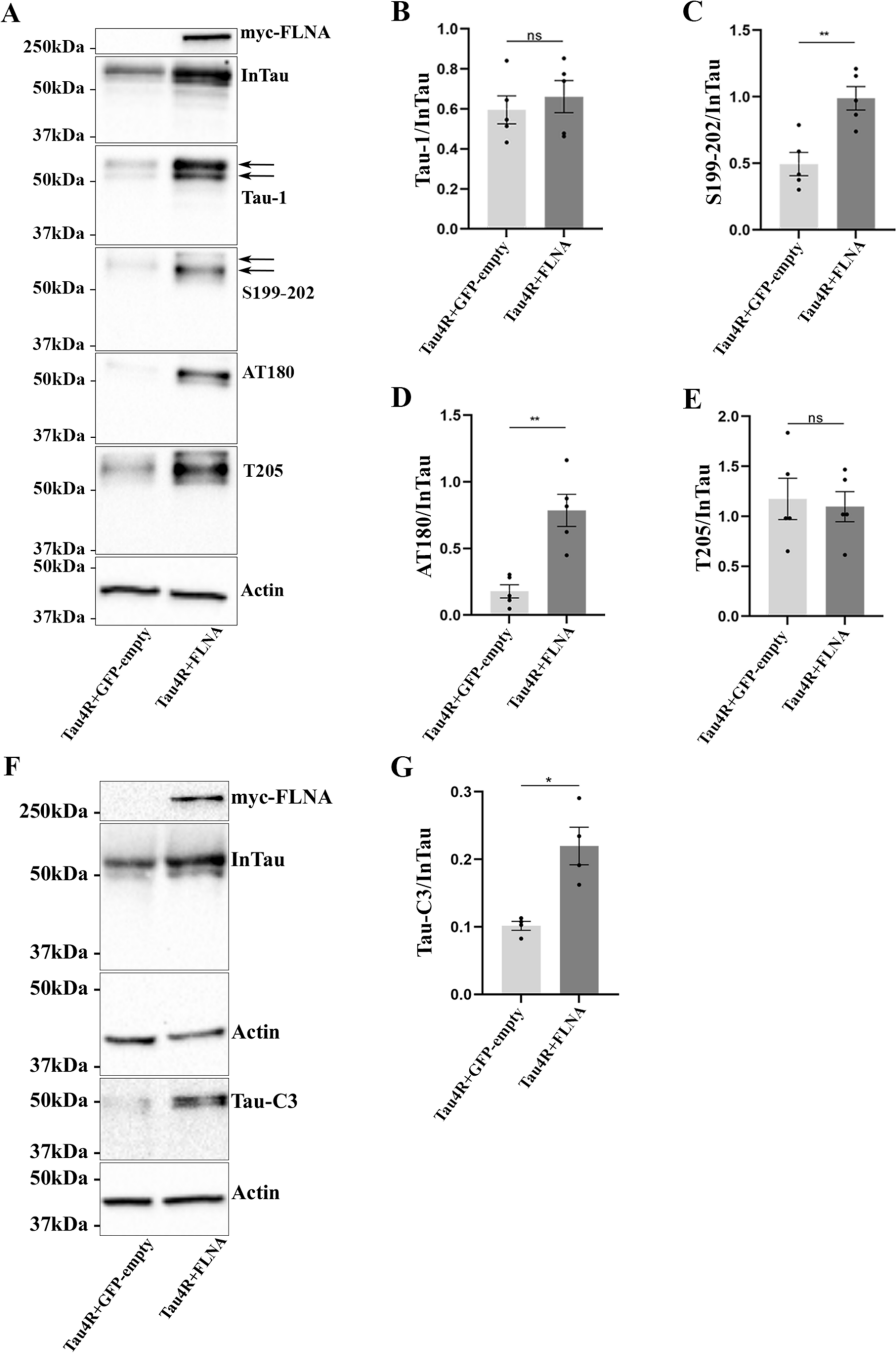
The overexpression of FLNA increases Tau phosphorylation and cleavage by caspase-3. N2a cells were transfected with either Flag-4RTau and EGFP-empty plasmids or Flag-4RTau and myc-FLNA for 48 h. **A** Western blotting analysis of the cell lysate with the antibody A0024 (InTau), the antibody Tau-1 recognizing unphosphorylated Tau at 195, 198, 199, and 202 a.a., the antibody recognizing Tau phosphorylated at S199-202, the antibody AT180 recognizing Tau phosphorylated at T231 and S235, and the antibody directed against Tau phosphorylated at T205. **B** Densitometry analysis of Tau-1 signal of InTau; *n* = 5. **C** Densitometry analysis of S199-202 signal of InTau; *n* = 5. **D** Densitometry analysis of AT180 signal of InTau; *n* = 5. **E** Densitometry analysis of T205 signal of InTau; *n* = 5. **F** Western blotting analysis of the cell lysate with the antibody Tau-C3 recognizing Tau cleaved by caspase-3. **G** Densitometry analysis of Tau-C3 signal of InTau; *n* = 4. Actin was used as a loading reference and an antibody directed against the myc-tag was used to evaluate the protein levels of myc-FLNA. Each phospho-Tau antibody signal and Tau-C3 signal were normalized with InTau signal. Data represent scatter plot and mean ± SEM. Each dot corresponds to a *n*. *p < 0.05; **p < 0.01; ***p < 0.001

Previous studies reported that FLNA could directly or through Aβ peptide alter Tau phosphorylation [28, 34]. Based on this, we explored whether Tau phosphorylation was increased by the overexpression of FLNA in N2a cells using four anti-phospho-Tau antibodies. The anti-Tau antibody Tau-1 that recognizes Tau when it is dephosphorylated at the epitope 195–202 showed no significant difference upon the overexpression of FLNA compared to Tau alone (Fig. 5A, B). Two of the three anti-phospho-Tau antibodies revealed an increase of Tau phosphorylation upon the overexpression of FLNA. The anti-phospho-Tau S199-202 antibody and anti-phospho-Tau AT180 antibody (T231/S235 residues), but not the anti-phospho-Tau T205 antibody, showed a significant increase by western blotting (Fig. 5C–E). In contrast, Tau overexpression did not result in increased FLNA phosphorylation levels (Fig. S1). Collectively, these results revealed that Tau phosphorylation was increased upon FLNA overexpression in N2a cells as reported in HEK 293 cells [34].

We also examined whether the interaction of Tau with FLNA significantly increased its cleavage by caspase-3 and its aggregation in N2a cells. Tau cleavage by caspase-3 was significantly increased when FLNA was overexpressed (Fig. 5F, G). Tau aggregation was measured using the Homogeneous Time Resolved Fluorescence (HTRF) Tau aggregation kit from CisBio. No increase of Tau aggregation was noted upon FLNA overexpression. The signal was at the background levels in cells overexpressing either Tau alone or Tau and FLNA (data not shown).

### Increase of Insoluble FLNA in AD Parietal Cortex but no Correlation with Insoluble Tau

**Fig. 6:**
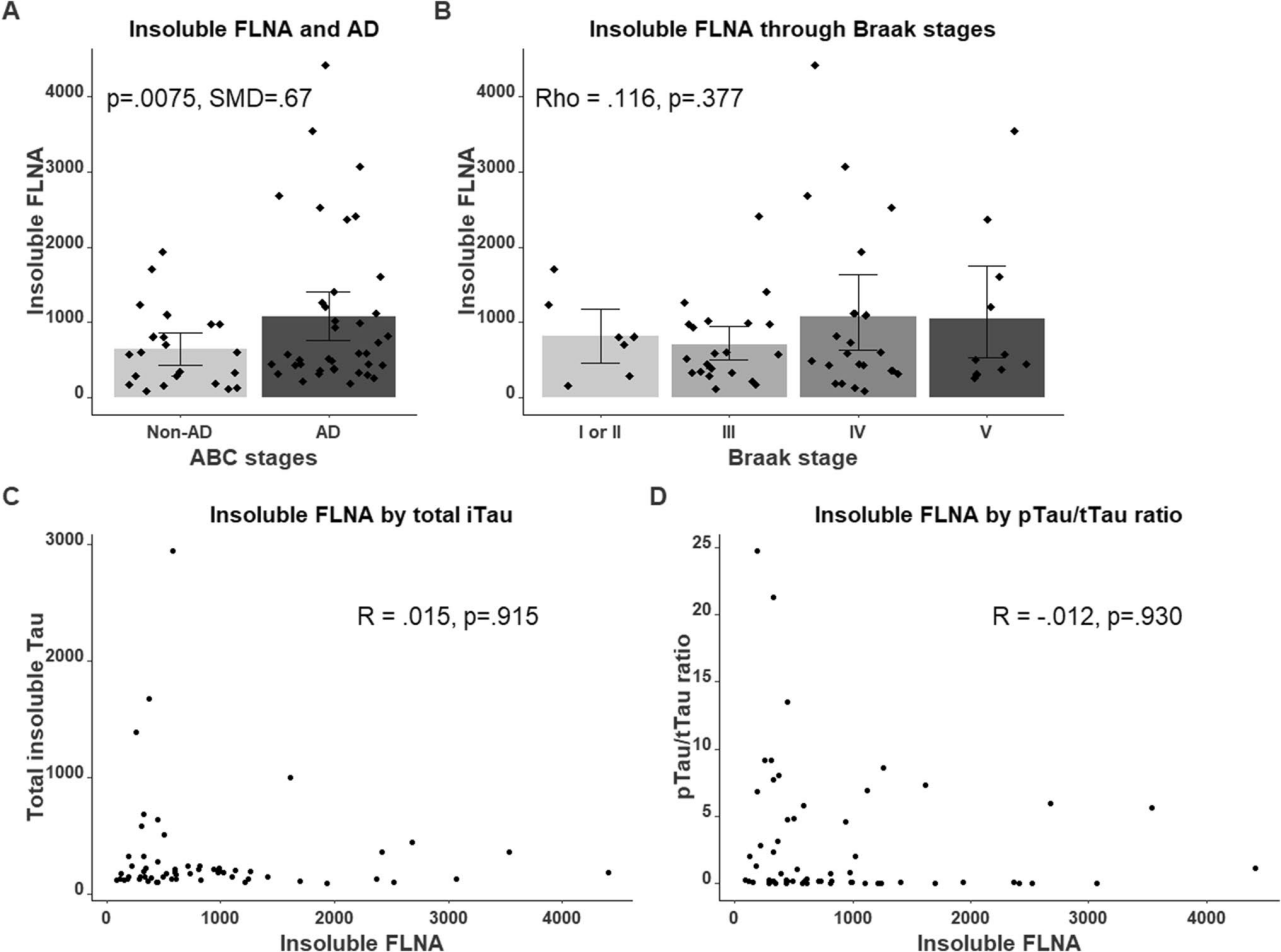
Insoluble FLNA is increased in AD but does not correlate with Tau pathology in the parietal cortex. **A** Patients were classified with the ABC scoring method of AD neuropathology. Higher insoluble FLNA levels were found in AD patients. **B** FLNA concentrations were not significantly associated with Braak stages. **C**, **D** Absence of correlation between insoluble FLNA and both insoluble total Tau (tTau) and the ratio of insoluble phosphorylated Tau (pTau) over tTau. For display purposes, Braak stages 1 and 2 were merged due to the small number of subjects

We examined the protein levels of insoluble FLNA in post-mortem anterior parietal cortex samples from 20 subjects presenting no cognitive deficits (NCI), 20 subjects with mild cognitive impairment (MCI), and 20 subjects with dementia [43]. The ABC coding system was used for the neuropathological diagnosis of AD [40, 44]. Higher levels of insoluble FLNA were observed in subjects with AD, compared to age-matched controls (Fig. 6A). No significant change of FLNA protein levels was observed in the soluble fraction (data not shown). In those samples of the parietal cortex, no correlation was observed between insoluble total and phosphorylated Tau and insoluble FLNA (Fig. 6B, C). Consistent with this, no association was noted between insoluble FLNA and the Braak stages used to assess Tau aggregation upon neuropathological examination (Fig. 6D). Although the accumulation of insoluble FLNA was not associated with post-mortem evidence of Tau pathology in this brain region, it does not exclude the possibility that FLNA could contribute to Tau pathology at earlier stages of the disease when Tau is still soluble.

### Binding of Tau to Microtubules and F-actin Was not Prevented by FLNA Overexpression in N2a Cells

**Fig. 7:**
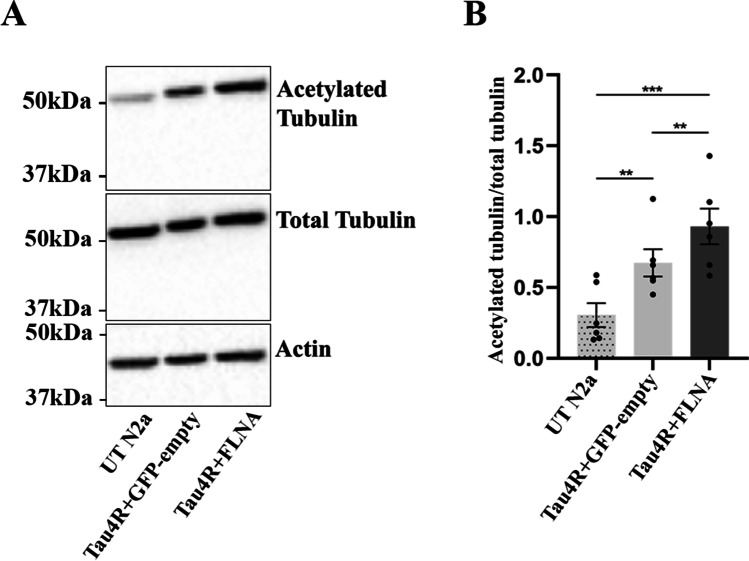
An increase of acetylated tubulin was found upon FLNA and Tau overexpression. N2a cells were co-transfected with either Flag-4RTau (Tau4R) and EGFP-empty plasmids or Flag-4RTau and myc-FLNA plasmids for 48 h. **A** Western blotting analysis of the cell lysate with the antibody directed against acetylated tubulin. Untransfected N2a cells (UT N2a) were used as a control. Actin was used as a loading reference. Acetylated tubulin was normalized with the total tubulin signal. The samples used in this experiment are the same ones that were used to measure endogenous Tau upon FLNA overexpression (Fig. 4C) and to measure FLNA phosphorylation (S1). **B** Densitometry analysis of the ratio acetylated tubulin/total tubulin signals revealed a significant increase of acetylated tubulin upon FLNA and Tau overexpression, *n* = 6. **p < 0.01; ***p < 0.001

**Fig. 8:**
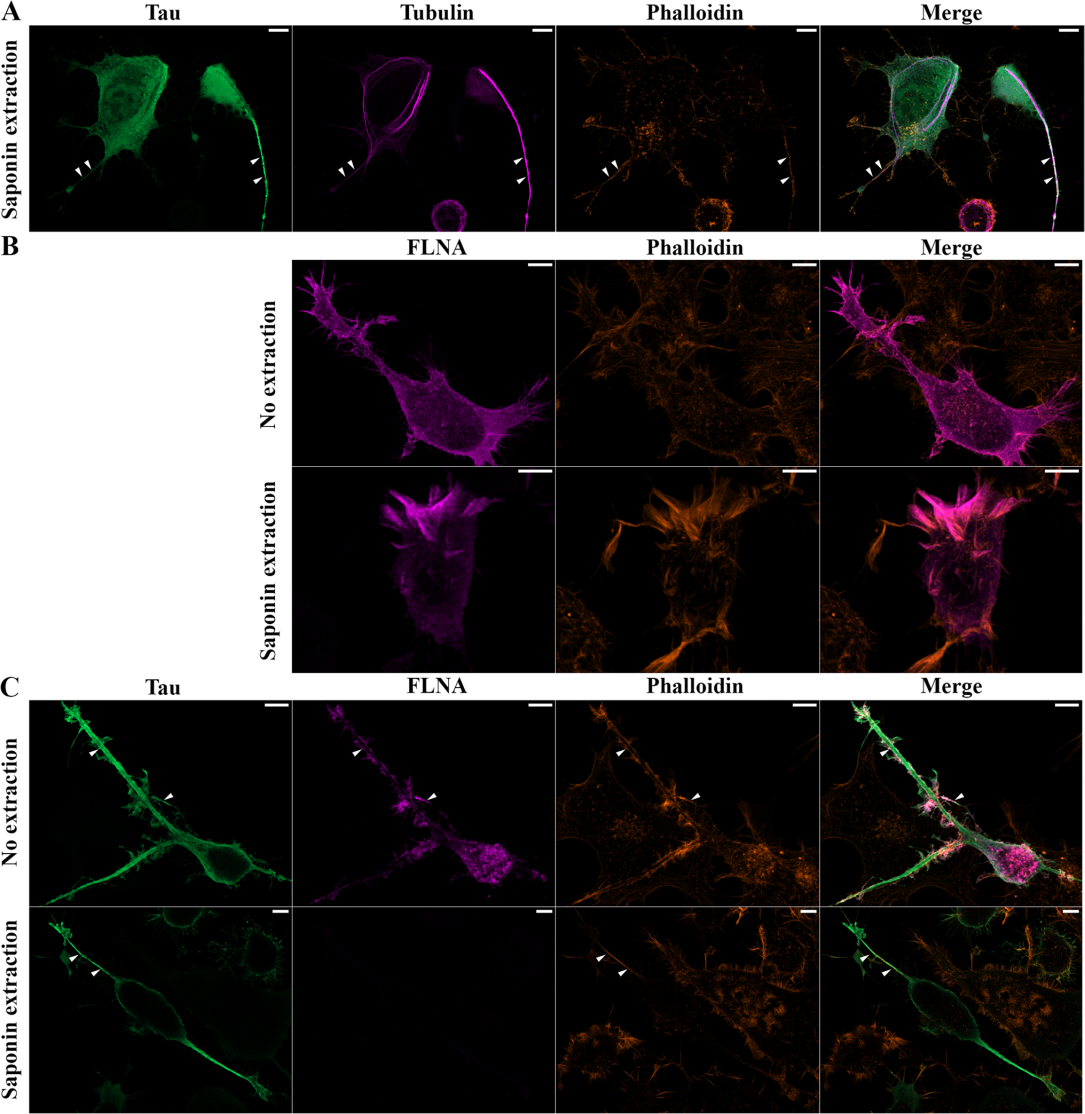
Binding of Tau to microtubules and F-actin upon FLNA overexpression. N2a cells were co-transfected with either Flag-4RTau (Tau4R) and EGFP-empty plasmids or Flag-4RTau and myc-FLNA plasmids for 48 h and then fixed and processed for immunofluorescence. **A** Co-localization of Tau with microtubules (tubulin) and F-actin (phalloidin) in the processes (white arrowheads) induced by its overexpression in saponin-extracted N2a cells. **B** Co-localization of FLNA with F-actin (phalloidin) in saponin-extracted and non-extracted cells. **C** Both Tau and FLNA were found on F-actin (white arrowheads) in non-extracted cells overexpressing FLNA and Tau. In extracted cells, no FLNA staining was detected upon the overexpression of FLNA and Tau. Tau was co-localized with phalloidin staining (white arrowheads). *n* = 3 Scale bars: 5 μm

Tau is known to stabilize microtubules which is revealed by an increase of acetylated tubulin upon its overexpression [47]. To determine whether Tau phosphorylation observed upon FLNA overexpression could affect its binding to microtubules, the amount of acetylated tubulin was examined by western blotting in N2a cells overexpressing either Tau alone or FLNA and Tau. As illustrated in Fig. 7 A and B, an increase of acetylated tubulin was noted when Tau alone was overexpressed, but this increase was significantly higher upon overexpression of FLNA and Tau. This was consistent with the increase of intracellular Tau following FLNA overexpression. The binding of Tau to microtubules was also monitored by confocal microscopy. To do so, the co-localization of Tau with microtubules was examined in N2a cells that were extracted with saponin before fixation. During the extraction, cytosolic Tau not attached to the cytoskeleton was washed out. Tau overexpression in N2a induced formation of processes emerging from the cell body (Fig. 8A). Tau binding to microtubules was confirmed by its co-localization with tubulin staining along the processes in extracted and fixed cells (Fig. 8A). As noted in other cellular models, Tau was also found on F-actin in extracted and fixed N2a cells transfected with either Tau alone or FLNA and Tau as revealed by the co-localization of Tau staining and phalloidin conjugated to Alexa-648 fluor dye, a toxin that specifically binds to F-actin [48]. Furthermore, F-actin was relocated in the Tau-induced processes. We then examined the co-localization of FLNA with F-actin in cells overexpressing either FLNA alone or FLNA and Tau. In non-extracted and extracted cells, overexpressed FLNA was found on F-actin as revealed by its co-localization with phalloidin (Fig. 8B). In cells overexpressing Tau and FLNA that were not extracted, both FLNA and Tau were found on F-actin, FLNA presenting the most important co-localization (Fig. 8C). Surprisingly, in contrast to cells overexpressing FLNA alone, no FLNA staining was detected in extracted cells overexpressing FLNA and Tau. This could indicate that FLNA binding to F-actin was weakened upon Tau accumulation and did not resist to the extraction procedure. Another possibility would be that the adhesion of the cells overexpressing FLNA and Tau was compromised making them detachable during the extraction procedure. Any of the above possibilities strongly suggests that FLNA interaction with F-actin might be modified upon Tau accumulation in N2a cells. The above results revealed that FLNA did not prevent Tau binding to microtubules and F-actin but that the binding of FLNA to F-actin might be altered by Tau accumulation.

### FLNA Induces the Accumulation of Annexin A2, a Tau Partner, in N2a Cells

**Fig. 9:**
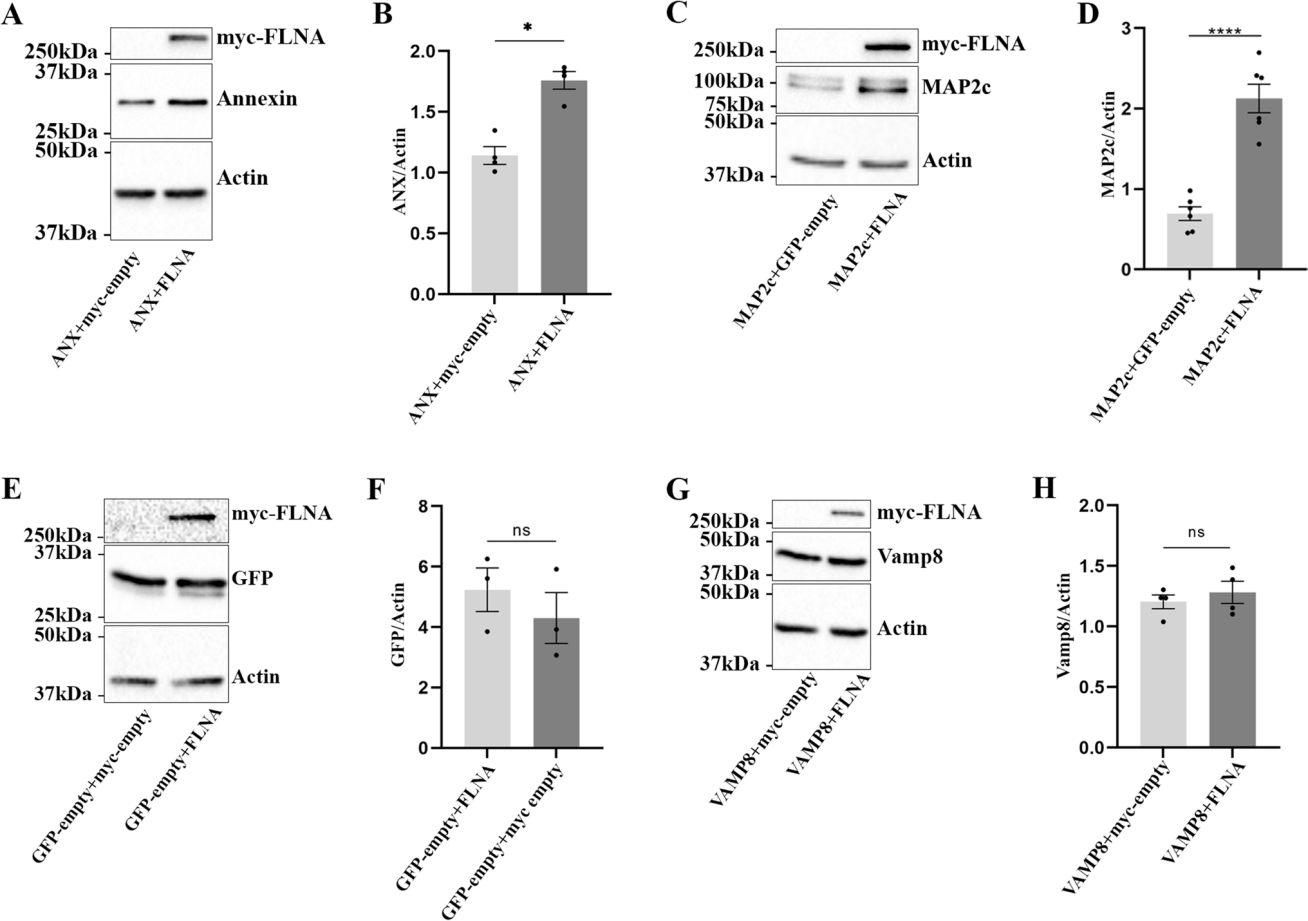
FLNA induces the accumulation of annexin A2 in N2a cells. GFP-annexin A2, GFP-VAMP8, GFP-MAP2c, and EGFP-empty plasmids were co-transfected with either myc-empty or myc-FLNA plasmids in N2a cells for 48 h. **A** Western blotting analysis of the cell lysate with the annexin A2 antibody. **B** Densitometry analysis of the annexin signal revealed a significant accumulation upon the overexpression of FLNA, *n* = 4. **C** Western blotting analysis of the cell lysate with the GFP antibody revealed an increase of the intracellular MAP2c upon the overexpression of FLNA. **D** Densitometry analysis of the GFP-MAP2c signal revealed a significant accumulation upon the overexpression of FLNA, *n* = 6. **E** Western blotting analysis of the cell lysate with the GFP antibody upon the overexpression of FLNA. **F** Densitometry analysis of the GFP signal revealed no change of GFP protein levels, *n* = 3. **G** Western blotting analysis of the cell lysate with the GFP antibody revealed no change of VAMP8 levels upon the overexpression of FLNA. **H** Densitometry analysis of the GFP-VAMP8 signal, *n* = 4. Actin was used as a loading reference and the antibody directed against the myc-tag was used to confirm the expression of myc-FLNA. Each of the above protein signal was normalized to the actin signal. Data represent scatter plot and mean ± SEM. Each dot corresponds to a *n*. *p < 0.05; **p < 0.01; ****p < 0.0001

We verified whether the effects of FLNA overexpression were specific to Tau or could also be observed for other proteins known to interact with it. To do so, annexin A2, a protein known to interact with FLNA, was overexpressed in N2a cells [36]. As noted for Tau, an increase of annexin A2 was observed upon FLNA overexpression (Fig. 9A, B). Interestingly, annexin A2 interacts with Tau and was shown to be involved in its axonal localization [37, 38]. Based on this, one can conclude that FLNA could contribute to Tau pathology by affecting its partners.

We also examined whether FLNA would induce the accumulation of MAP2c, a MAP found in the dendrites and axon during brain development, that shares 80% sequence homology with Tau [49–51]. Our GFP-trap and Myc-trap experiments revealed that the domain of Tau interacting with FLNA was located between the 157 and 383 a.a. where the microtubule-binding domain is found and indicated that MAP2c might also interact with FLNA. GFP-tagged MAP2c was co-transfected with myc-FLNA in N2a cells. The antibody GFP showed an increase of total MAP2c signal in the cell lysate upon overexpression of FLNA compared to cells overexpressing MAP2c alone (Fig. 9C, D). These data indicated that MAPs other than Tau could be affected by FLNA. To further demonstrate that FLNA only induced the accumulation of its interactors, we examined the effects of FLNA on proteins that are not known to interact with it such as GFP and VAMP8, a SNARE involved in the fusion of late endosomes with the plasma membrane (Fig. 9E–H) [52]. No accumulation of these two proteins was observed in N2a upon FLNA overexpression. The above results indicated that the increased protein levels of FLNA observed in AD brain could lead to the accumulation of its interactors including Tau and annexin A2.

## Discussion

Previous studies have reported a link between FLNA and Tau pathology [28, 34]. In the present study, we confirmed the interaction of Tau with FLNA in N2a cells. This interaction was mediated by a domain located in 157–383 a.a. of Tau. We observed that the overexpression of FLNA resulted in the intracellular accumulation of wild-type Tau as previously reported and also that of Tau mutants (P301L, V337M, and R406W) linked to FTLD-Tau. Tau phosphorylation and its cleavage by caspase-3 but not its aggregation were increased upon FLNA overexpression in N2a cells. In the parietal cortex of AD brain, insoluble FLNA concentration was increased compared to control brain but it did not correlate with insoluble Tau. Interestingly, FLNA overexpression did not prevent the binding of Tau to microtubules and F-actin. However, FLNA binding to F-actin might be modified upon Tau overexpression. Lastly, our results revealed that FLNA also induced the accumulation of the Tau interacting partner annexin A2 as well as MAP2c, a MAP sharing sequence homology with Tau. Collectively, our data indicated that in Tauopathies, FLNA could contribute to Tau pathology by acting directly on Tau and indirectly through its interacting partners.

In neurons, FLNA is found in the SD compartment [53]. When Tau is redistributed in this compartment in Tauopathies, our results and those of Tsujikawa et al. indicate that Tau could interact with FLNA [34]. Tau can be divided in four domains: the N-terminal projecting at the surface of microtubules, the proline-rich region located between the N-terminal and the microtubule-binding domain, the microtubule-binding domain containing either 3 or 4 repeats, and the C-terminal region [54]. Our results revealed that the domain of Tau mediating its interaction with FLNA is located in its C-terminal half containing the microtubule-binding domain and the C-terminal region. In the human brain, Tau presents 6 isoforms, 3 isoforms with 3 repeats in the microtubule-binding domain called 3R, and 3 isoforms with 4 repeats in the microtubule-binding domain called 4R [54]. Each of the 3R and 4R isoforms contains either no insert in the N-terminal called 0N3R and 0N4R, one insert called 1N3R and 1N4R or two inserts called 2N3R and 2N4R. FLNA increased the intracellular accumulation of both 0N4R and 0N3R Tau isoforms in N2a cells, indicating that the interacting domain of Tau with FLNA is comprised in the sequence common to these isoforms. This means that the 2 inserts in the N-terminal found in the 2N3R and 2N4R isoforms and the second repeat in the microtubule-binding domain found in the 4R isoforms are not involved in Tau interaction with FLNA. We also noted an accumulation of MAP2c upon FLNA overexpression in N2a cells indicating that FLNA could interact with it. This implies that the interacting domain of Tau with FLNA is contained in the sequence common to MAP2c located in its microtubule-binding domain and the C-terminal end region [49–51]. We also examined whether FLNA would lead to an accumulation of the Tau mutants P301L, V337M, and R406W in N2a cells. P301L is located in the second repeat of the microtubule-binding domain only found in 4R isoforms, V337M in the fourth repeat of the microtubule-binding domain found in all Tau isoforms, and R406W in the C-terminal also found in all Tau isoforms [1]. The accumulation of these mutants in N2a cells upon FLNA overexpression strongly indicates that these mutants can interact with FLNA and that Tau interacting domain most likely does not involve these mutated amino acids.

In a recent study, it was demonstrated that FLNA increased Tau protein levels through direct interaction mediated by F-actin in neurons [34]. A similar mechanism could exist in N2a cells where both FLNA and Tau were found to co-localize with F-actin. We also observed that the protein levels of annexin A2 and MAP2c, both known to interact and reorganize F-actin, were increased in N2a upon FLNA overexpression [55, 56]. This increase was not noted for proteins that did not interact with F-actin and FLNA. FLNA, Tau, annexin A2, and MAP2c can reorganize F-actin [55–57]. A recent study reported that the reorganization of F-actin impacted mRNA translation. F-actin can serve as a platform where mRNA can be in a repressed state that is relieved upon F-actin reorganization [58]. In such a scenario, one can speculate that the reorganization of F-actin by FLNA could allow the Tau, annexin A2, and MAP2c mRNA repressed state to be relieved. Another possibility would be that the reorganization of F-actin by FLNA allowed the transport of Tau, annexin A2, and MAP2c mRNA along F-actin to translationally active sites.

Another explanation for the accumulation of Tau upon FLNA overexpression could be that Tau was less degraded in the presence of FLNA. This scenario is consistent with previous studies reporting that FLNA can stabilize proteins by interacting with them, which impairs their degradation [22, 59]. Tau can be degraded by both the proteasome and via autophagy [60]. In both cases, Tau ubiquitination is necessary [61, 62]. In N2a cells, Tau was still ubiquitinated upon FLNA overexpression (unpublished data) indicating that other factors could impair Tau degradation in cells overexpressing FLNA. Furthermore, we did not observe any significant change of the autophagic markers LC3 and p62 in N2a cells overexpressing FLNA (unpublished data). One possibility could be that in the SD compartment, FLNA would impair the interaction of Tau with proteins known to shuttle it either to the proteasome (p62, BAG2, CHIP, HSP70, and HSP90) or to autophagy (BAG3 and NDP52) [60, 63]. Based on a recent study, this possibility is unlikely because an increase of Tau binding to HSP90, HSP70, HSP40, and ubiquitin was observed in HEK293 overexpressing FLNA [34]. All the above observations indicate that Tau accumulation upon FLNA overexpression did not result from an impairment of its degradation.

Tau protein undergoes several post-translational modifications, phosphorylation being the main one. Tau contains 85 phosphorylation sites. In AD, 45 of these sites were shown to be phosphorylated. Upon FLNA overexpression, Tau phosphorylation was increased at certain sites in N2a cells as previously reported in HEK293 and mouse brain [34]. The phosphorylation of 5 sites known to be phosphorylated in AD was examined in cells overexpressing FLNA [64]. Interestingly, we found that the phosphorylation of S199/S202 sites was significantly increased in the presence of FLNA as opposed to that of T205, which was unchanged in N2a cells. It was showed that the phosphorylation of the different sites varies between neuronal compartments. For example, Tau phosphorylated at S199 and S202 is enriched in the SD compartment whereas Tau phosphorylated at T205 is preferentially found in the axon [65]. Based on our data, one can conclude that the interaction of Tau with FLNA could contribute to this differential phosphorylation state of Tau between the SD compartment and the axon. The increase of Tau phosphorylation upon FLNA overexpression at certain sites could indicate that when Tau is included in protein complex containing FLNA it modifies its conformation and/or accessibility to either kinases or phosphatases. Because we noted an increase of Tau phosphorylation, we examined whether the phosphorylation of FLNA was increased. Phosphorylation of FLNA at S2152 prevents its cleavage by calpain and affects the interaction with its binding partners [66–69]. The antibody directed against phosphorylated FLNA showed no difference in the phosphorylation levels of either endogenous or transfected FLNA in N2a cells. This could indicate that different kinases phosphorylated Tau and FLNA in N2a cells or that FLNA and Tau compete for the same kinases, Tau being the best substrate for them. We also noted an increased cleavage of Tau by caspase-3 upon the overexpression of FLNA. The mechanisms leading to this modification remain to be elucidated. Collectively, the interaction of Tau with FLNA could favor post-translational modifications that increases its toxicity. Consistent with this, it was reported that the FLNA ortholog in *Drosophila* modulates Tau toxicity [70, 71].

In contrast to recent data demonstrating that FLNA drives Tau aggregation, we did not observe an increase of Tau aggregation upon FLNA overexpression in N2a cells [34]. Tau phosphorylation state could contribute to the lack of Tau aggregation in these cells. Upon FLNA overexpression, Tau phosphorylation at S199/S202 and T231/S235 was increased. Interestingly, Dujardin et al. revealed that the phosphorylation of T231/S235 was positively correlated with AD clinical progression and Tau seeding capacity [72]. In the same study, phosphorylation of Tau at S198/S199/S202 was shown to negatively correlate with Tau seeding activity. These observations indicate that upon FLNA overexpression, the phosphorylation of Tau at S199/S202 could negate the seeding activity of Tau phosphorylated at T231/S235 in N2a cells. The lack of Tau aggregation by FLNA in N2a cells can also be explained by technical reasons. Firstly, FLNA and Tau were only co-expressed for 48 h, which could be insufficient to induce Tau aggregation in N2a cells. Secondly, we used a Tau aggregation kit that can detect Tau dimers and oligomers as well as Tau aggregates. However, Tau oligomer levels might have been lower than the detection threshold of this kit. Thirdly, Tsujikawa K et al. reported that Tau aggregation by FLNA was proportional to its protein levels [34]. A significant increase of Tau protein levels and aggregation was only noted with the highest FLNA protein levels. It is possible that in N2a cells, FLNA protein levels did not reach the threshold necessary to induce Tau aggregation. Fourthly, the cellular context could be determinant in FLNA capacity to induce Tau aggregation.

In the present study, we observed that the accumulation of Tau upon the overexpression of FLNA was independent of Tau isoforms in N2a cells as reported in mouse brain [34]. In Tauopathies, different combinations of isoforms are found in Tau aggregates [1]. Aggregates predominantly composed of 4R isoforms are found in cortical basal degeneration (CBD) and progressive supranuclear palsy (PSP), whereas aggregates predominantly composed of 3R isoforms are formed in Pick disease. In AD, Tau aggregates contain both 3R and 4R isoforms. All the above observations indicate that if FLNA was involved in Tau aggregation, all isoforms should be found in Tau aggregates as noted in AD. However, the contribution of FLNA to Tau aggregation in Tauopathies remains controversial. In a previous study, FLNA was found to co-localize with Tau aggregates in AD, FTLD-Tau, Pick’s disease, and PSP using immunocytochemistry [32]. The presence of FLNA in Tau aggregates and its increased protein levels in AD were further confirmed by using MS [23, 33]. In the study of Tsujikawa et al., no increase of FLNA protein levels and no co-localization of FLNA with NFTs were found in the frontal cortex of AD patients [34]. In our study, no correlation was found between insoluble FLNA and insoluble Tau in the parietal cortex of AD patients. The effects of FLNA on Tau could have occurred at earlier stages of the disease in this brain region, not detectable by simple post-mortem correlative analysis in AD patients. For example, in this region, FLNA could solely contribute to the increased protein levels of Tau at early stages of the disease before aggregation takes place. All these discrepancies could merely be explained by the fact that the contribution of FLNA to Tau aggregation could depend on the brain region. In such a scenario, the contribution of FLNA to Tau aggregation could involve co-factors that are differently expressed in the brain regions affected by Tau pathology. These co-factors would be involved in determining which isoform aggregates. It was recently reported that early- and late-aggregating proteins drive Aβ aggregation. Some of these proteins such as lamin A/C, ubiquitin-like modifier-activating enzyme-1, and 14–3-3 proteins were also found in Tau aggregates, suggesting that Tau aggregation could also involve early- and late-aggregating proteins [73].

Upon FLNA overexpression, proteins other than Tau also accumulated in N2a cells. This is the case of annexin A2, a partner of Tau. Annexin A2 was reported to be involved in the axonal localization of Tau and to participate in its redistribution to the SD compartment in Tauopathies [38]. If FLNA is increased in AD as previously reported, this could lead to the accumulation of annexin A2, which could alter the axonal localization of Tau. This indicates that FLNA can indirectly contribute to Tau pathology through Tau partners. We also noted an accumulation of MAP2c upon FLNA overexpression in N2a cells. MAP2 was shown to compensate for Tau suppression in mice [74]. MAP2c belongs to the MAP2 family also comprising MAP2a/b, important MAPs that stabilize microtubules in the dendrites [51]. Any alteration of these MAP protein levels could have detrimental consequences on dendritic morphology and functioning by affecting microtubule dynamics.

All the above observations indicate that more work needs to be done before elaborating therapeutic strategies that target FLNA for preventing Tau pathology. For example, it is possible that co-factors are necessary for FLNA to induce Tau pathology. The identification of these co-factors might help to reconcile the discrepancies between Tauopathies and brain regions. Lastly, the contribution of FLNA to Tau pathology and its involvement in Aβ pathology through PS1 and α7nAChR suggest that FLNA could be part of the convergent pathway inducing Tau aggregation and phosphorylation in both sporadic and familial AD [26–28].

## Supplementary Information

Below is the link to the electronic supplementary material.

Supporting Fig. 1 (PNG 195 kb)

High resolution image (TIF 17324 kb)

## Data Availability

Our manuscript has no associated data.
