# Supplementary figures and images for: Direct and Indirect Effects of Filamin A on Tau Pathology in Neuronal Cells
Source: Mol Neurobiol. 2022 Nov 18;60(2):1021–39. doi: 10.1007/s12035-022-03121-w (PMC9849303; doi:10.1007/s12035-022-03121-w)

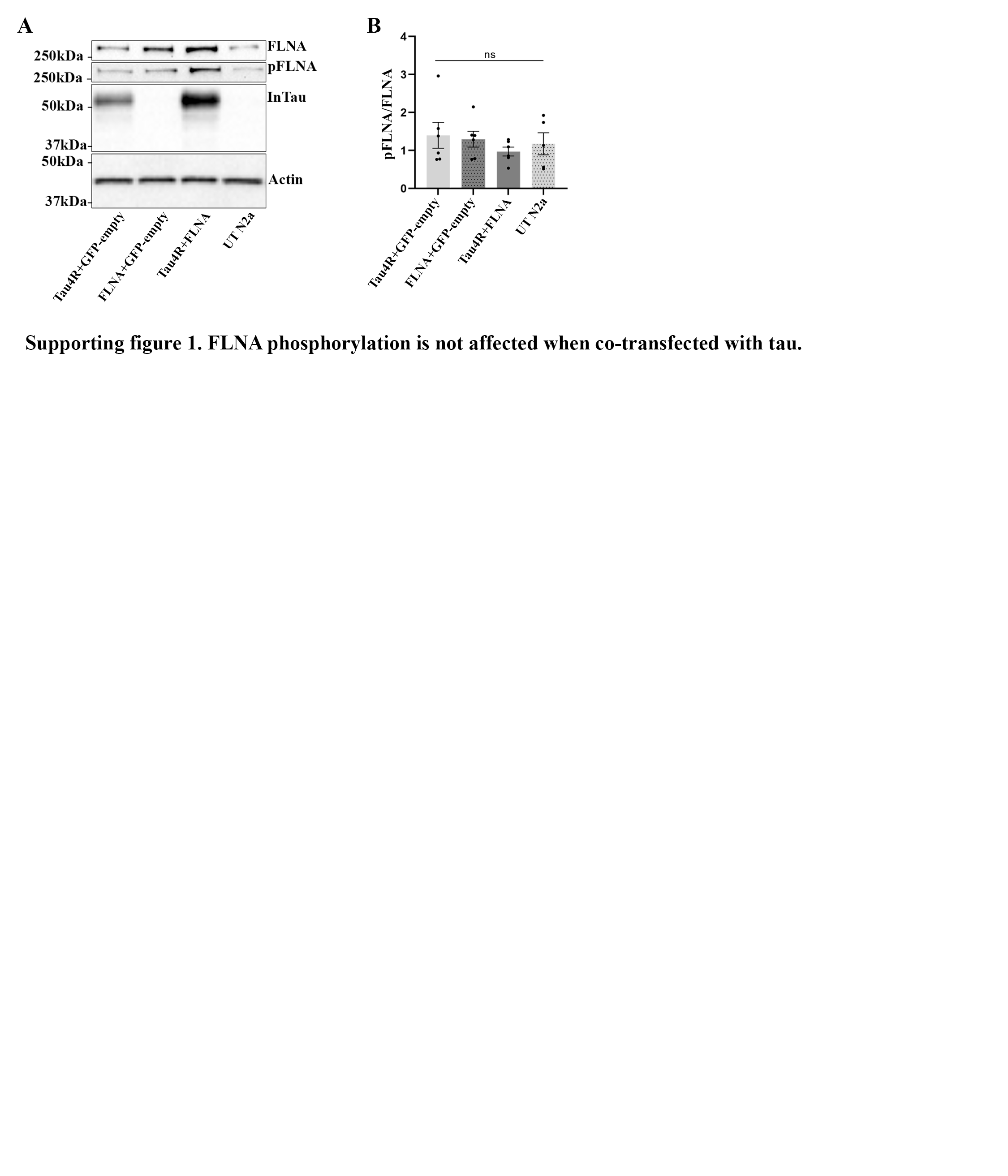

Supplement: Supplementary file 1 — (PNG 195 kb) [file 12035_2022_3121_Fig10_ESM.png]

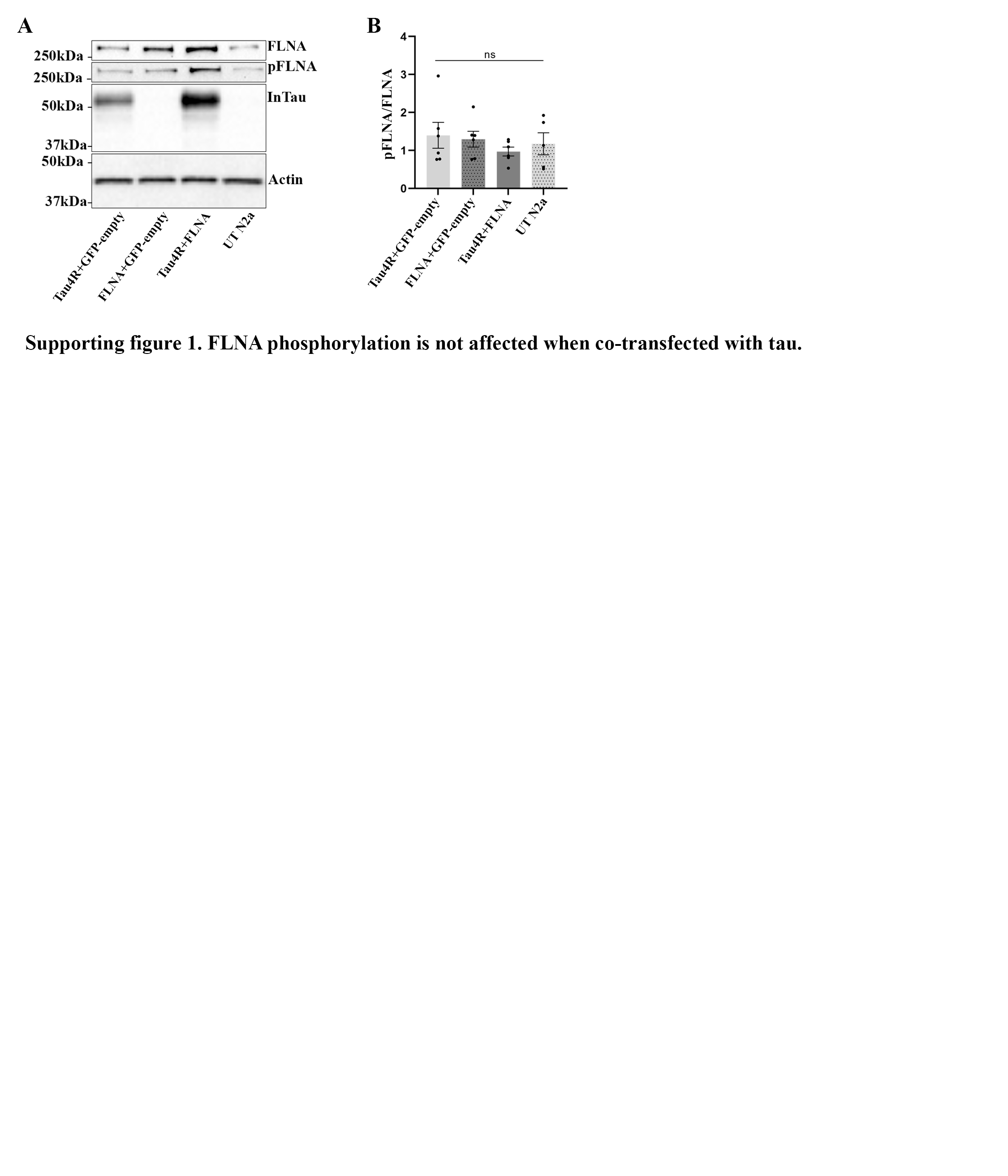

Supplement: Supplementary file 2 — High resolution image (TIF 17324 kb) [file 12035_2022_3121_MOESM1_ESM.tif]
